# Cervical Cancer Genetic Susceptibility: A Systematic Review and Meta-Analyses of Recent Evidence

**DOI:** 10.1371/journal.pone.0157344

**Published:** 2016-07-14

**Authors:** Gabriela A. Martínez-Nava, Julián A. Fernández-Niño, Vicente Madrid-Marina, Kirvis Torres-Poveda

**Affiliations:** 1 Synovial Fluid Laboratory, Instituto Nacional de Rehabilitación “Luis Guillermo Ibarra” (INR LGII), Mexico City, Mexico; 2 Public Health Department, School of Medicine, Universidad Industrial de Santander, Bucaramanga, Colombia; 3 Universidad Manuela Beltrán, Bucaramanga, Colombia; 4 Chronic Infectious Diseases and Cancer Division, Center for Research on Infectious Diseases, Instituto Nacional de Salud Pública (INSP), Cuernavaca, Morelos, Mexico; 5 CONACYT Research Fellow, Instituto Nacional de Salud Pública (INSP), Cuernavaca, Morelos, Mexico; West China Second Hospital, Sichuan University, CHINA

## Abstract

**Introduction:**

Cervical cancer (CC) has one of the highest mortality rates among women worldwide. Several efforts have been made to identify the genetic susceptibility factors underlying CC development. However, only a few polymorphisms have shown consistency among studies.

**Materials and Methods:**

We conducted a systematic review of all recent case-control studies focused on the evaluation of single nucleotide polymorphisms (SNPs) and CC risk, stringently following the “PRISMA” statement recommendations. The MEDLINE data base was used for the search. A total of 100 case-control studies were included in the meta-analysis. Polymorphisms that had more than two reports were meta-analyzed by fixed or random models according to the heterogeneity presented among studies.

**Results:**

We found significant negative association between the dominant inheritance model of *p21 rs1801270* polymorphism (C/A+A/A) and CC (pooled OR = 0.76; 95%CI: 0.63–0.91; *p*<0.01). We also found a negative association with the *rs2048718 BRIP1* polymorphism dominant inheritance model (*T/C+C/C*) and CC (pooled OR = 0.83; 95%CI: 0.70–0.98; *p* = 0.03), as well as with the *rs11079454 BRIP1* polymorphism recessive inheritance model and CC (pooled OR = 0.79; 95%CI: 0.63–0.99; *p* = 0.04). Interestingly, we observed a strong tendency of the meta-analyzed studies to be of Asiatic origin (67%). We also found a significant low representation of African populations (4%).

**Conclusions:**

Our results provide evidence of the negative association of *p21* rs1801270 polymorphism, as well as BRIP1 rs2048718 and rs11079454 polymorphisms, with CC risk. This study suggests the urgent need for more replication studies focused on GWAS identified CC susceptibility variants, in order to reveal the most informative genetic susceptibility markers for CC across different populations.

## Introduction

Even though it is the cancer with the greatest demonstrated potential for secondary prevention, cervical cancer (CC) constitutes a significant public health problem. It has the third highest cancer incidence rate in women worldwide and even the second highest in some developing countries, like Mexico [1,2]. In 2014, CC was the second cause of death by malignant tumors in women from Mexico, with an incidence rate of 6.08 per 100 000 women over 10 years, corresponding to 3, 063 new cases [3].

According to records from Globocan 2012 and assuming that the estimated CC incidence rates remained constant in Mexico, an alarming 28% increase in CC cases in this region is estimated by the year 2020 [4]. Globally, combined strategies of HPV vaccination and HPV-based screening tests could theoretically control CC in any population in which a large coverage with both preventive options is ensured. However, accessibility of developing countries to vaccination and low-cost HPV screening options are at present the barriers to overcome [5]. Currently in Mexico, CC primary prevention policy dictates the administration of the vaccine against HPV infection in girls aged 9 to 13 years within the national immunization program. Secondary prevention of CC is achieved through pap smears screening, and molecular detection of high-risk HPV in women aged 25 to 64 years of age, which has substantially improved the sensitivity of detection of precancerous lesions with cytology alone [6,7]. This has led to the search for new primary or secondary CC prevention alternatives, complementing existing programs. In that sense, efforts have mainly focused on the search for genetic susceptibility factors to enable an early identification of women at higher CC risk, and on generating evidence for the identification of those women who should be directed without delay to more robust and stringent prevention programs, thereby achieving greater impact in the reduction of CC mortality.

Although several factors that contribute to CC development have been identified—mainly intrinsic factors (genetic), and extrinsic factors belonging to the Human Papillomavirus (HPV)—genetic factors show great potential for use as susceptibility or prognosis factors [8,9]. Most association studies for CC worldwide evaluate single nucleotide polymorphisms (SNP) in candidates’ genes involved in oncogenesis and cellular immune response, as there is plenty of evidence showing the existence of an immune response evasion in patients with persistent HPV infection and CC [10–12]. However, few of the studied polymorphisms in this neoplasm have shown consistent associations, and largely, the magnitude of the association is small [13].

Therefore, we formulated the following PICO (participants of interest, intervention, control and primary outcome of interest) question: Which SNPs reported in literature confer susceptibility or protection to CC in the international consensus? To address it we conducted a systematic review and meta-analyses following “PRISMA” statement recommendations (S1 File) [14].

## Materials and Methods

### Search Strategy

A systematic literature search led by the PICO question was conducted to identify articles covering SNPs associated with the risk of developing cervical cancer (CC). From this question, a search in the MEDLINE database was performed through the PubMed database browser with the combination of the following terms: “polymorphism”, “single nucleotide polymorphism”, “SNP”, “uterine cervical neoplasm”, “cervical intraepithelial neoplasia” and “cervical cancer” from November 2009 to February 2015. The search was limited to the studies published 2009 onwards to ensure homogenization in the genotyping technique used across studies. The search was performed with the restriction of language of the full-text to English, Spanish or Portuguese. The scope of computerized literature search was expanded according to the reference lists of retrieved articles. The original articles were screened manually by two independent reviewers (TPK and MNGA). If the full text of an article was not available online to perform the screening we proceed to contact the first author of the article by e-mail requesting a copy of their work.

### Study Selection

Studies concerning the association of SNPs with the risk of developing CC were included if the following conditions were met: (i) any study describing the association of SNPs with CC; (ii) genotypes or allelic frequencies reported for control and case groups, given that for the pooled ORs calculation this data is strictly needed; (iii) case-control or nested case-control studies, since other epidemiological designs might result in heterogeneous case definitions; (iv) any study that reported the numbers of both controls and CC cases; (v) include at least 100 cases, in order to include only does studies with the minimal number of cases necessary to have a representation of all three genotypes (assuming that the minor allele frequency is of at least 1%); (vii) not focused on HLA antigen genetic markers. The latter selection criteria is due to the high concordance among studies over HLA haplotypes association with CC, and the aim of the present work is to evaluate the association of CC with genetic polymorphisms that remains to be fully accepted as genetic susceptibility factors for the disease (which is not the case of HLA studied haplotypes).

Since our event of interest was CC risk all selected studies should defined as main outcome CC at any stage (carcinoma *in situ*, invasive cervical cancer, cervical adenocarcinoma or cervix cancer). Those studies evaluating only the association between precancerous lesions (cervical intraepithelial neoplasia 1–3, low grade and/or high grade squamous intraepithelial lesions) were excluded, as precancerous lesions do not always lead to cancer development. In the case that a study assessed the association with both precancerous lesions and cervical cancer, only the group of cases defined as histologically confirm CC were taken into account, if the study did not differentiate between precancerous lesion and CC patients the study was excluded. This criteria also applied for those studies assessing other types of cancer besides CC in the same report.

Of those studies that fulfilled the afore mention selection criteria the main genotyping techniques used were TaqMan probes, high resolution melting, Ilumina GoldenGate multiplex assays, restriction length fragments polymorphism and DNA sequencing (S1 Table).

### Data Extraction

The following data was extracted from each article: authors; year of publication; country; ethnicity of participants (categorized as Caucasians, Asians, Latinos, etc.); study design; number of controls and cases; genotyping method; distribution of age, gene name, polymorphism and genotypic frequency. The data was extracted and registered into two databases by two reviewers (TPK and MNGA) independently. The reviewers were blind to journal names, institutions or fund grants. Any discrepancy between these two expert investigators in the field was resolved by a third reviewer (MMV), who also extracted the data and participated in the discussion with them and made the final decision.

### Statistical Analysis

Statistical analyses were performed for each gene polymorphism for which at least two studies were available. First, Odds Ratios (OR), with their respective confidence intervals (CI), were estimated by individual study based on the primary data obtained from the review.

Pooled ORs were estimated for each polymorphism and Χ^2^ type tests for heterogeneity between and intra-study, known as Cochran’s *Q*, were performed. The null hypothesis for this test asserts that the association is the same in each study and therefore the observed variability is explained by chance alone [15]; in contrast, the alternative hypothesis proposes that heterogeneity does exist in the association between studies. Derived from the previous calculation, the I^2^ was estimated [15], which is a function of Cochran’s Q and measures the grade of heterogeneity. In this study, the grade of heterogeneity was considered low if the I^2^ was between 25 and 50%, moderate if the I^2^ was between 50 and 75%, and high if the I^2^ was greater than 75% [16]. In order to have more robust standards for heterogeneity evaluation, especially given the low sample size for each meta-analysis and the subsequent potential type II errors, a Τ^2^ test was also applied, whose null hypothesis and interpretation is equivalent to the Cochran’s *Q*.

As a stringent approach to decide if the random effects model, rather than the fixed effects model, was used for considering the weights in the pooled OR estimation for each polymorphism, the afore mentioned Cochran’s *Q* or the Τ^2^ were also considered and not only the I^2^. In that sense, if any of the two former measures yielded a significant result with an α of 0.05 (which as expected is always equivalent to an at least moderate I^2^), then random effects models were applied. In the case in which both tests were not statistically significant (equivalent to a low I^2^) fixed effects models were used.

Sensibility analyzes were carried out for the polymorphisms with more than four studies by iteratively removing a study to repeatedly recalculate the pooled OR, and finding consistency in all cases. Finally, potential publication bias was evaluated with an Egger Test only in polymorphisms with more than five studies regardless of whether significant association was found in the meta-analysis [17,18].

For all the analyses a statistical significance α level of 0.05 was considered. The α level was set at this value as this is the most widely used in the majority of the original studies of the field. Furthermore, the definition of the significance level was made taking into consideration the limitation of the statistical power of the meta-analysis performed for each association in order to avoid a possible type 2 error [19]. Nevertheless, in all the associations the obtained p-value is reported, and not only if the association was or was not significant for their direct conceptual interpretation under the critical judgment of the reader [20].

All analyses were performed in STATA v.14 (StataCorporation, CollegeStation, TX, USA).

## Results

**Fig 1:**
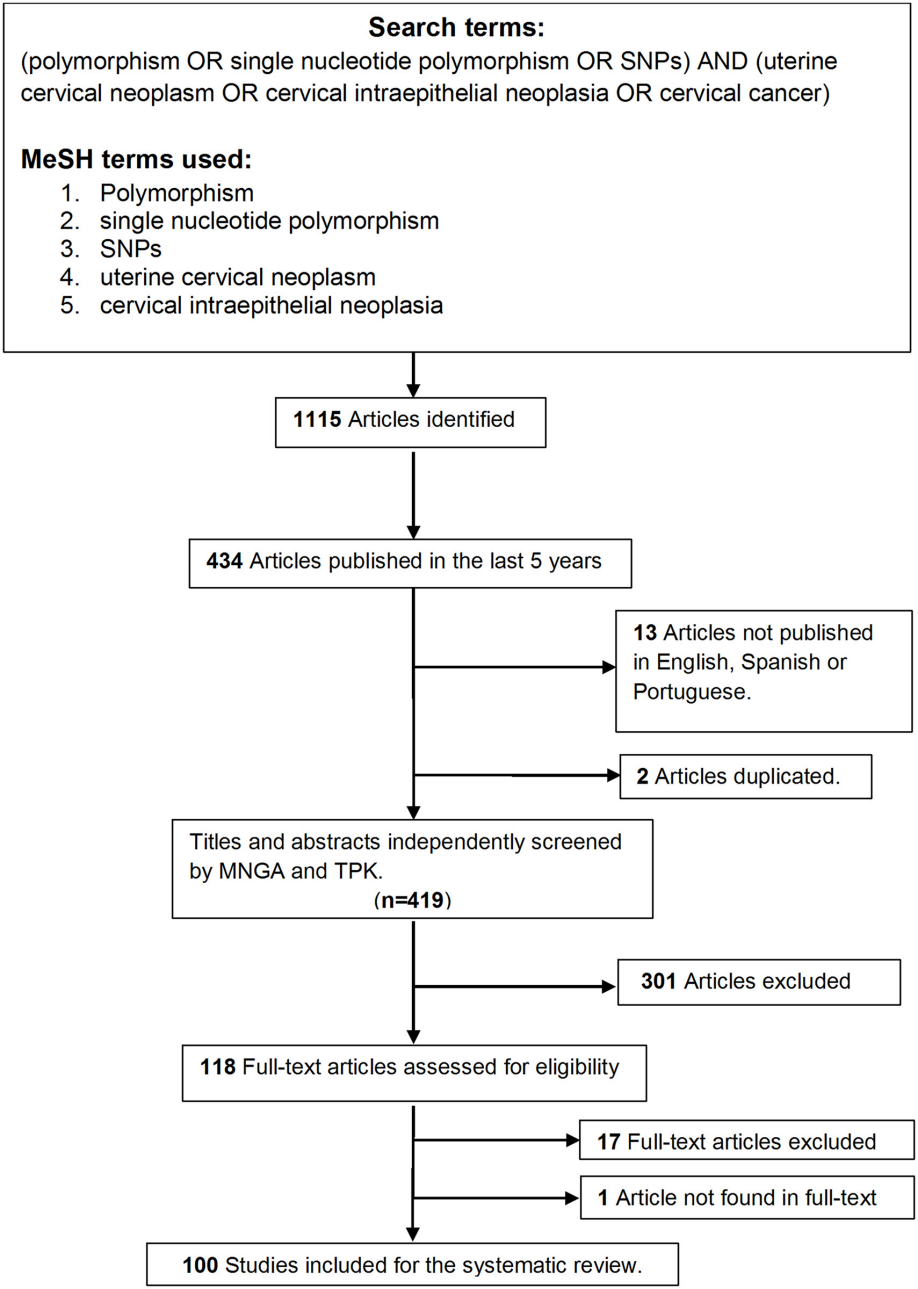
Study selection for inclusion in meta-analysis flowchart.

In the search for eligible studies, we inputted the aforementioned keywords into MEDLINE and obtained a total of 1115 studies. When we applied the filters for language and year of publication we ended up with 419 studies to be screened. Only 118 studies met the criteria specified in the method section to be further assessed in full-text. Of these, 22 articles were excluded since they did not report genotypic frequencies or had a sample size of less than 100 CC cases (S2 Table). We were not able to obtain one article in full text. In total, 100 studies were included in the analysis from which 17 SNPs were meta-analyzed for codominant, dominant and recessive inheritance models, and 21 SNPs for the allelic model (Fig 1, S1 Table).

In the 100 studies reviewed, 636 SNPs of 151 different genes were reported, with 86.95% of these SNPs being reported in only one study (p50 = 1 an interquartile range = 0). All the meta-analyzed SNPs were reported in at least three different studies, with 42.86% SNPs reported in more than three studies. The *p53 rs1042522* polymorphism was the most reported with seven studies in total (S3 Table).

For each SNP, the pooled OR and 95% CI for the heterozygous genotype, minor allele homozygous genotype, dominant inheritance model (heterozygous genotype and minor allele homozygous genotype vs. ancestral allele homozygous genotype), recessive inheritance model (minor allele homozygous genotype vs. heterozygous and ancestral allele homozygous genotype), and for the minor allele were calculated. In all models the heterogeneity between studies was taken into account when selecting which meta-analysis model to apply (random effects or fixed effects model).

**Fig 2:**
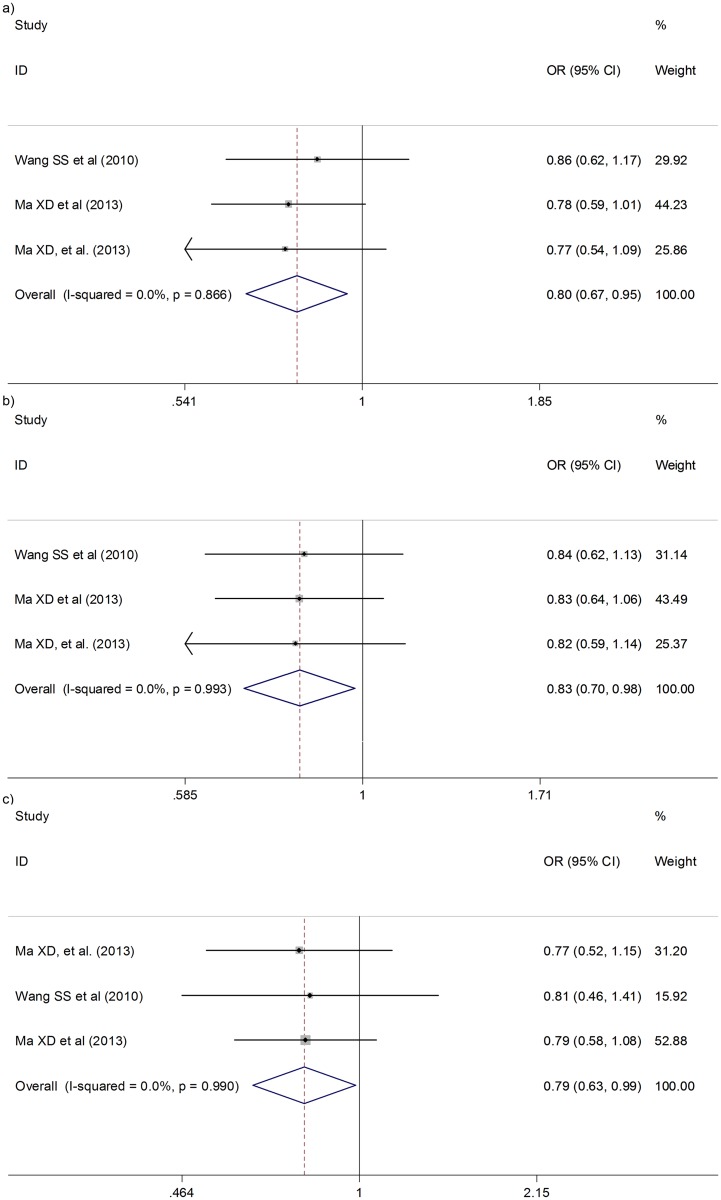
Forest plot for cervical cancer risk for heterozygous genotype (a) and dominant inheritance model (b) of rs2048718 BRIP1 gene polymorphism; and for recessive inheritance model (c) of rs11079454 BRIP1 gene polymorphism.

**Fig 3:**
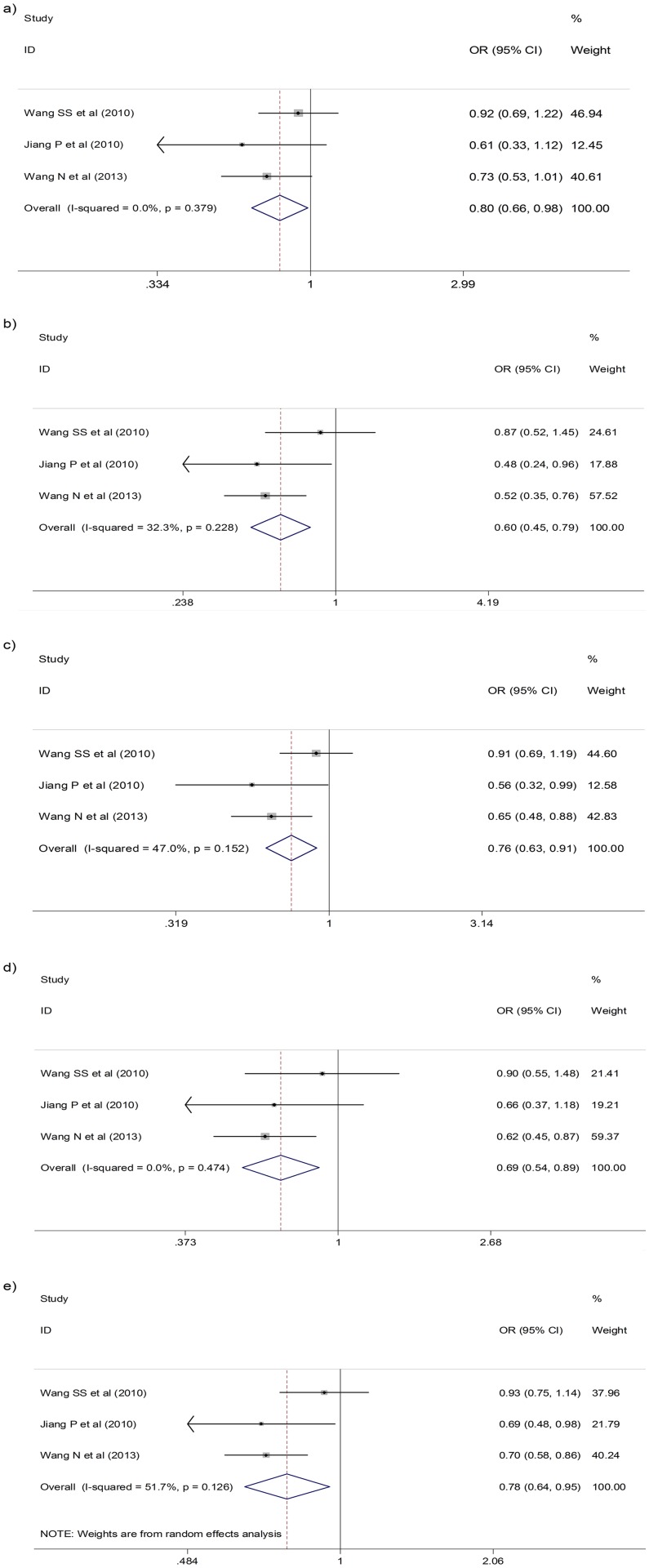
Forest plot for cervical cancer risk for heterozygous (a) and minor allele homozygous (b) genotypes, dominant (c) and recessive (d) inheritance models, and minor allele (e) of rs1801270 p21 gene polymorphism.

The meta-analysis for the heterozygous genotype showed that two SNPs were negatively associated with CC risk: the *rs2048718 BRIP1* gene polymorphism (pooled OR = 0.80; 95%CI: 0.67–0.95; *p* = 0.01) (Fig 2a) and the *rs1801270 p21* gene polymorphism (pooled OR = 0.80; 95%CI: 0.66–0.98; *p* = 0.03) (Fig 3a). Both of these SNPs did not present heterogeneity between studies (p-value for heterogeneity>0.05; I^2^ = 0.00 and Tau^2^ = 0.00) so the fixed effects model was used (Table 1).

**Table 1:** Meta-analysis for heterozygous genotypes of each SNP.

SNP	Gene	Model	Study	OR (95%CI)	Weight (%)	Cochran’s Q	I^2^	Τ^2^	pooled OR (95%CI)	p-value
rs11079454	BRIP1	Fixed	Ma XD et al (2013)	1.15 (0.86–1.53)	38.5	0.44	0.00	0.00	1.04 (0.87–1.24)	0.69
			Wang SS et al (2010)	0.89 (0.67–1.19)	38.70					
			Ma XD et al (2013)	1.12 (0.77–1.64)	22.46					
rs2048718	BRIP1	Fixed	Wang SS et al (2010)	0.86 (0.62–1.17)	29.92	0.87	0.00	0.00	**0.80 (0.67–0.95)**	**0.01**
			Ma XD et al (2013)	0.78 (0.59–1.01)	44.23					
			Ma XD et al (2013)	0.77 (0.54–1.09)	25.86					
rs6504074	BRIP1	Random	Ma XD et al (2013)	**0.74 (0.56–0.96)**	36.93	0.09	59.3	0.04	0.86 (0.65–1.14)	0.29
			Wang SS et al (2010)	1.14 (0.84–1.55)	33.32					
			Ma XD et al (2013)	0.76 (0.54–1.08)	29.75					
rs231775	CTLA-4	Random	Xioing YH et al (2014)	1.24 (0.92–1.68)	28.11	**0.04**	77.7	0.13	0.87 (0.58–1.30)	0.49
			Gokhale P et al (2013)	**0.45 (0.24–0.83)**	18.65					
			Wang SS et al (2010)	1.18 (0.85–1.64)	27.38					
			Hu L et al (2010)	**0.68 (0.46–0.99)**	25.86					
rs5742909	CTLA-4	Fixed	Gokhale P et al (2013)	1.01 (0.34–3.00)	9.89	0.06	63.4	0.06	1.27 (0.88–1.85)	0.21
			Ivansson EL et al (2010)	1.05 (0.84–1.33)	48.07					
			Xioing YH et al (2014)	**1.66 (1.22–2.27)**	42.04					
rs1800872	IL-10	Random	Wang SS et al (2010)	0.92 (0.69–1.22)	46.28	0.12	52.8	0.05	0.78 (0.54–1.13)	0.19
			Singhal P et al (2014)	**0.56 (0.37–0.84)**	35.87					
			Shekari M et al (2012)	1.00 (0.48–2.09)	17.86					
rs1800896	IL-10	Random	Wang SS et al (2010)	0.94 (0.71–1.26)	29.99	**0.02**	68.9	0.09	1.34 (0.93–1.92)	0.12
			Wang Q et al (2011)	1.50 (0.98–2.30)	24.34					
			Barbisan G et al (2012)	1.16 (0.72–1.89)	22.21					
			Singhal P et al (2014)	**2.11 (1.34–3.31)**	32.46					
rs16944	IL-1B	Random	Wang SS et al (2010)	1.02 (0.74–1.40)	31.97	**0.05**	62.6	0.08	1.05 (0.74–1.51)	0.77
			Al-Tahhan MA et al (2011)	0.54 (0.26–1.15)	14.98					
			Qian N et al (2010)	**1.56 (1.12–2.17)**	31.12					
			Zidi S et al (2014)	1.00 (0.59–1.70)	21.92					
rs1801133	MTHFR	Fixed	Kohaar I et al (2010)	1.03 (0.66–1.61)	33.71	0.92	0.00	0.00	0.96 (0.74–1.24)	0.74
			Tong SY et al (2011)	0.94 (0.62–1.43)	38.00					
			Mostowska A et al (2011)	0.90 (0.55–1.46)	28.29					
rs11515	p16	Random	Thakur N et al (2012)	0.32 (0.18–0.59)	30.09	**<0.01**	84.3	0.29	0.75 (0.38–1.46)	0.39
			Vargas-Torres et al (2014)	1.03 (0.67–1.59)	34.22					
			Wang SS et al (2010)	1.12 (0.78–1.61)	35.69					
rs3088440	p16	Random	Wang SS et al (2010)	1.11 (0.83–1.49)	52.87	0.08	61.3	0.11	1.16 (0.69–1.94)	0.57
			Vargas-Torres et al (2014)	0.98 (0.64–1.51)	43.99					
			Thakur N et al (2012)	25.19 (1.48–429.73)	3.13					
rs1801270	p21	Fixed	Wang N et al (2013)	0.73 (0.53–1.01)	40.61	0.38	0.00	0.00	**0.80 (0.66–0.98)**	**0.03**
			Wang SS et al (2010)	0.92 (0.69–1.22)	46.94					
			Jiang P et al (2010)	0.61 (0.33–1.12)	12.45					
rs1042522	p53	Random	Jiang P et al (2010)	0.71 (0.33–1.54)	12.21	**0.04**	58.1	0.11	1.25 (0.88–1.77)	0.22
			Ferreira da Silva I et al (2010)	**1.92 (1.05–3.52)**	15.90					
			El khair MM et al (2010)	0.64 (0.34–1.20)	15.35					
			Yang SD et al (2014)	1.57 (0.84–2.94)	15.47					
			Ye F et al (2010)	**1.68 (1.26–2.23)**	25.27					
			Jiang P et al (2010)	1.18 (0.64–2.18)	15.79					
rs1136410	PARP-1	Random	Roszak A et al (2013)	**1.38 (1.03–1.86)**	32.18	**0.03**	72.6	0.05	1.03 (0.76–1.40)	0.86
			Ye F et al (2012)	1.01 (0.79–1.29)	35.25					
			Wang SS et al (2010)	0.78 (0.59–1.05)	32.58					
rs352140	TLR9	Random	Roszak A et al (2012)	1.37 (0.99–1.91)	34.46	**0.03**	67.1	0.14	1.22 (0.76–1.94)	0.41
			Lai ZZ et al (2013)	**6.93 (1.53–31.30)**	7.81					
			Zidi S et al (2014)	0.77 (0.46–1.29)	27.51					
			Pandey S et al (2011)	1.03 (0.66–1.60)	30.21					
rs1800629	TNF	Random	Barbisan G et al (2012)	0.45 (0.20–1.00)	11.78	**0.03**	66.2	0.07	1.17 (0.85–1.61)	0.34
			Ivansson EL et al (2010)	1.20 (0.97–1.47)	35.42					
			Roszak A et al (2015)	1.19 (0.88–1.62)	30.10					
			Sousa H et al (2014)	**1.80 (1.14–2.83)**	22.71					
rs25487	XRCC1	Random	Wang SS et al (2010)	0.87 (0.65–1.16)	41.44	0.07	62.6	0.07	1.09 (0.74–1.60)	0.67
			Roszak A et al (2011)	**1.57 (1.04–2.39)**	32.74					
			Settheetham-Ishida W (2011)	0.97 (0.57–1.68)	25.82					

A significant negative association was also observed between the minor allele homozygous genotype (*A/A*) carriers for the *rs1801270 p21* gene polymorphism and CC risk (pooled OR = 0.60; 95%CI: 0.42–0.86; *p*<0.01) (Fig 3b). However, the association between the *rs2048718 BRIP1* gene minor allele homozygous genotype (*C/C*) carriers and CC risk did not reach statistical significance (pooled OR = 1.05; 95%CI: 0.80–1.40; *p* = 0.82) (Table 2).

**Table 2:** Meta-analysis for minor allele homozygous genotypes of each SNP.

SNP	Gene	Model	Study	OR (95%CI)	Weight (%)	Cochran’s Q	I^2^	Τ^2^	pooled OR (95%CI)	p-value
rs11079454	BRIP1	Fixed	Ma XD et al (2013)	0.86 (0.60–1.23)	50.60	0.95	0.00	0.00	0.83 (0.65–1.07)	0.16
			Wang SS et al (2010)	0.77 (0.44–1.36)	19.84					
			Ma XD et al (2013)	0.83 (0.52–1.32)	29.56					
rs2048718	BRIP1	Fixed	Wang SS et al (2010)	0.80 (0.54–1.18)	55.52	0.36	2.7	**<0.01**	0.96 (0.72–1.30)	0.82
			Ma XD et al (2013)	1.23 (0.71–2.15)	28.37					
			Ma XD et al (2013)	1.22 (0.58–2.57)	16.11					
rs6504074	BRIP1	Fixed	Ma XD et al (2013)	0.89 (0.53–1.48)	30.36	0.48	0.00	0.00	1.05 (0.80–1.40)	0.71
			Wang SS et al (2010)	1.25 (0.84–1.84)	52.58					
			Ma XD et al (2013)	0.86 (0.43–1.71)	17.06					
rs231775	CTLA-4	Random	Xioing YH et al (2014)	**0.58 (0.36–0.92)**	25.27	**0.02**	71.0	0.13	0.71 (0.46–1.08)	0.11
			Gokhale P et al (2013)	**0.46 (0.23–0.92)**	18.12					
			Wang SS et al (2010)	1.22 (0.84–1.76)	28.37					
			Hu L et al (2010)	**0.65 (0.44–0.94)**	28.24					
rs5742909	CTLA-4	Random	Gokhale P et al (2013)	excluded		0.16	50.3	0.78	2.73 (0.52–14.41)	0.24
			Ivansson EL et al (2010)	1.44 (0.44–4.68)	63.09					
			Xioing YH et al (2014)	8.17 (0.98–68.34)	36.91					
rs1800872	IL-10	Random	Wang SS et al (2010)	1.19 (0.74–1.91)	34.99	**<0.01**	86.8	0.54	0.75 (0.31–1.85)	0.54
			Singhal P et al (2014)	**0.32 (0.20–0.54)**	34.43					
			Shekari M et al (2012)	1.15 (0.55–2.44)	30.58					
rs1800896	IL-10	Random	Wang SS et al (2010)	0.78 (0.42–1.46)	25.33	**<0.01**	90.1	0.97	1.78 (0.64–4.94)	0.27
			Wang Q et al (2011)	1.53 (0.79–2.95)	25.11					
			Barbisan G et al (2012)	1.24 (0.52–2.95)	23.32					
			Singhal P et al (2014)	**6.27 (3.80–10.35)**	26.24					
rs16944	IL-1B	Random	Wang SS et al (2010)	1.11 (0.75–1.64)	31.83	**0.02**	68.9	0.17	1.11 (0.67–1.84)	0.68
			Al-Tahhan MA et al (2011)	**0.26 (0.08–0.79)**	13.38					
			Qian N et al (2010)	1.46 (0.98–2.18)	31.49					
			Zidi S et al (2014)	1.78 (0.91–3.45)	23.30					
rs1801133	MTHFR	Fixed	Kohaar I et al (2010)	1.14 (0.30–4.34)	10.41	0.57	0.00	0.00	0.93 (0.60–1.42)	0.73
			Tong SY et al (2011)	1.04 (0.61–1.78)	65.27					
			Mostowska A et al (2011)	0.62 (0.26–1.48)	24.32					
rs11515	p16	Fixed	Thakur N et al (2012)	0.16 (0.01–3.32)	8.32	0.34	8.5	0.60	1.26 (0.52–3.06)	0.61
			Vargas-Torres et al (2014)	1.85 (0.54–6.37)	44.99					
			Wang SS et al (2010)	1.26 (0.37–4.22)	46.69					
rs3088440	p16	Random	Wang SS et al (2010)	0.86 (0.54–1.39)	45.09	**0.01**	78.2	1.57	2.31 (0.43–12.37)	0.33
			Vargas-Torres et al (2014)	1.29 (0.34–4.91)	36.07					
			Thakur N et al (2012)	**75.00 (3.78–1486.79)**	18.84					
rs1801270	p21	Fixed	Wang N et al (2013)	**0.52 (0.35–0.76)**	57.52	0.23	32.3	0.03	**0.60 (0.45–0.79)**	**<0.01**
			Wang SS et al (2010)	0.87 (0.52–1.45)	24.61					
			Jiang P et al (2010)	**0.48 (0.24–0.96)**	17.88					
rs1042522	p53	Random	Jiang P et al (2010)	1.54 (0.72–3.31)	15.84	**<0.01**	82.2	0.50	1.16 (0.62–2.18)	0.64
			Ferreira da Silva I et al (2010)	1.19 (0.58–2.44)	16.26					
			El khair MM et al (2010)	0.69 (0.30–1.56)	15.26					
			Yang SD et al (2014)	**2.17 (1.15–4.09)**	17.10					
			Ye F et al (2010)	**0.45 (0.30–0.67)**	19.09					
			Jiang P et al (2010)	**2.25 (1.11–4.54)**	16.45					
rs1136410	PARP-1	Random	Roszak A et al (2013)	1.68 (0.87–3.62)	29.53	**<0.01**	84.6	0.32	1.58 (0.78–3.19)	0.20
			Ye F et al (2012)	2.59 (1.79–3.73)	36.09					
			Wang SS et al (2010)	0.89 (0.57–1.40)	34.39					
rs352140	TLR9	Fixed	Roszak A et al (2012)	**1.48 (1.01–2.18)**	41.80	0.17	41.0	0.07	1.45 (0.95–2.22)	0.08
			Lai ZZ et al (2013)	7.92 (0.97–64.52)	3.85					
			Zidi S et al (2014)	1.75 (0.96–3.16)	28.36					
			Pandey S et al (2011)	0.90 (0.47–1.70)	25.99					
rs1800629	TNF	Random	Barbisan G et al (2012)	9.47 (0.48–185.22)	8.09	**0.03**	67.6	0.52	1.85 (0.72–4.72)	0.20
			Ivansson EL et al (2010)	0.78 (0.46–1.32)	38.58					
			Roszak A et al (2015)	2.42 (1.15–5.11)	34.31					
			Sousa H. et al (2014)	3.24 (0.64–16.28)	19.02					
rs25487	XRCC1	Random	Wang SS et al (2010)	**0.56 (0.34–0.92)**	37.97	**<0.01**	85.8	0.72	1.05 (0.36–3.05)	0.93
			Roszak A et al (2011)	**2.31 (1.33–4.01)**	37.22					
			Settheetham-Ishida W et al (2011)	0.84 (0.22–3.25)	24.80					

The *rs1801270 p21* gene polymorphism maintained the significant negative association previously observed for the heterozygous and the minor allele homozygous carriers when assessing the association by dominant (pooled OR = 0.76; 95%CI: 0.63–0.91; *p*<0.01) (Table 3 and Fig 3c) and recessive (pooled OR = 0.69; 95%CI: 0.54–0.89; *p*<0.01) inheritance models (Table 4 and Fig 3d). Additionally, the significant negative association between *rs2048718 BRIP1* gene polymorphism heterozygous carriers and CC risk was also observed for the dominant inheritance model (OR = 0.83; 95%CI: 0.70–0.98; *p* = 0.03) (Table 3 and Fig 2b). Although statistically significant association with this polymorphism was not detected in the recessive inheritance model, we identified a borderline negative association with a different *BRIP1* gene polymorphism (rs11079454) showing a *p* value of 0.04 (Table 4 and Fig 2c).

**Table 3:** Meta-analysis for dominant inheritance model of each SNP.

SNP	Gene	Model	Study	OR (95%CI)	Weight (%)	Cochran’s Q	I^2^	Τ^2^	pooled OR (95%CI)	p-value
rs11079454	BRIP1	Fixed	Ma XD et al (2013)	1.05 (0.80–1.38)	39.20	0.61	0.00	0.00	0.98 (0.82–1.16)	0.78
			Wang SS et al (2010)	0.88 (0.67–1.15)	38.09					
			Ma XD et al (2013)	1.03 (0.72–1.46)	22.70					
rs2048718	BRIP1	Fixed	Wang SS et al (2010)	0.84 (0.62–1.13)	31.14	0.99	0.00	0.00	**0.83 (0.70–0.98)**	**0.03**
			Ma XD et al (2013)	0.83 (0.64–1.06)	43.49					
			Ma XD et al (2013)	0.82 (0.58–1.14)	25.37					
rs6504074	BRIP1	Random	Ma XD et al (2013)	0.76 (0.59–0.98)	36.54	0.06	63.6	0.04	0.88 (0.67–1.17)	0.38
			Wang SS et al (2010)	1.17 (0.87–1.56)	33.44					
			Ma XD et al (2013)	0.78 (0.56–1.08)	30.03					
rs231775	CTLA-4	Random	Xioing YH et al (2014)	1.06 (0.79–1.41)	28.18	**<0.01**	76.5	0.11	0.83 (0.57–1.20)	0.32
			Gokhale P et al (2013)	**0.45 (0.25–0.81)**	18.40					
			Wang SS et al (2010)	1.20 (0.88–1.62)	27.65					
			Hu L et al (2010)	**0.66 (0.46–0.94)**	25.76					
rs5742909	CTLA-4	Random	Gokhale P et al (2013)	1.01 (0.34–3.00)	10.89	**0.04**	68.0	0.07	1.30 (0.87–1.93)	0.20
			Ivansson EL et al (2010)	1.06 (0.85–1.34)	47.13					
			Xioing YH et al (2014)	**1.72 (1.27–2.34)**	41.99					
rs1800872	IL-10	Random	Wang SS et al (2010)	0.96 (0.74–1.27)	39.64	**0.01**	78.9	0.16	0.77 (0.45–1.30)	0.33
			Singhal P et al (2014)	**0.48 (0.32–0.70)**	35.88					
			Shekari M et al (2012)	1.07 (0.52–1.18)	24.48					
rs1800896	IL-10	Random	Wang SS et al (2010)	0.92 (0.70–1.21)	26.69	**<0.01**	88.4	0.29	1.51 (0.86–2.64)	0.15
			Wang Q et al (2011)	1.50 (1.00–2.25)	24.85					
			Barbisan G et al (2012)	1.17 (0.74–1.87)	23.79					
			Singhal P et al (2014)	**3.30 (2.18–5.00)**	24.67					
rs16944	IL-1B	Random	Wang SS et al (2010)	1.04 (0.78–1.40)	30.64	**0.02**	70.7	0.10	1.05 (0.71–1.53)	0.82
			Al-Tahhan MA et al (2011)	**0.46 (0.23–0.93)**	16.75					
			Qian N et al (2010)	**1.53 (1.12–2.09)**	29.97					
			Zidi S et al (2014)	1.16 (0.70–1.93)	22.64					
rs1801133	MTHFR	Fixed	Kohaar I et al (2010)	1.04 (0.67–1.60)	32.41	0.82	0.00	0.00	0.96 (0.75–1.22)	0.71
			Tong SY et al (2011)	0.97 (0.66–1.43)	39.83					
			Mostowska A et al (2011)	0.85 (0.53–1.35)	27.76					
rs11515	p16	Random	Thakur N et al (2012)	**0.31 (0.17–0.56)**	30.29	**<0.01**	86.3	0.33	0.75 (0.37–1.51)	0.42
			Vargas-Torres et al (2014)	1.05 (0.69–1.59)	34.22					
			Wang SS et al (2010)	1.16 (0.81–1.66)	35.49					
rs3088440	p16	Random	Wang SS et al (2010)	1.06 (0.80–1.40)	53.01	0.07	62.5	0.11	1.14 (0.69–1.90)	0.61
			Vargas-Torres et al (2014)	1.00 (0.66–1.52)	43.93					
			Thakur N et al (2012)	**27.17 (1.59–463.161)**	3.06					
rs1801270	p21	Fixed	Wang N et al (2013)	**0.65 (0.48–0.88)**	42.83	0.2	47.0	0.03	**0.76 (0.63–0.91)**	**<0.01**
			Wang SS et al (2010)	0.91 (0.69–1.19)	44.60					
			Jiang P et al (2010)	**0.56 (0.32–0.99)**	12.58					
rs1042522	p53	Fixed	Jiang P et al (2010)	1.06 (0.52–2.17)	10.62	0.17	35.0	0.04	1.28 (0.98–1.66)	0.07
			Ferreira da Silva I et al (2010)	1.69 (0.94–3.04)	14.31					
			El khair MM et al (2010)	0.65 (0.36–1.19)	13.74					
			Yang SD et al (2014)	**1.83 (1.03–3.24)**	14.81					
			Ye F et al (2010)	1.27 (0.97–1.68)	31.76					
			Jiang P et al (2010)	1.46 (0.83–2.60)	14.75					
rs1136410	PARP-1	Random	Roszak A et al (2013)	**1.42 (1.07–1.88)**	32.42	**0.01**	77.0	0.06	1.11 (0.81–1.53)	0.51
			Ye F et al (2012)	1.20 (0.95–1.53)	34.81					
			Wang SS et al (2010)	0.80 (0.61–1.06)	32.78					
rs352140	TLR9	Random	Roszak A et al (2012)	**1.41 (1.03–1.92)**	33.14	**0.02**	70.1	0.14	1.38 (0.87–2.17)	0.17
			Lai ZZ et al (2013)	**7.26 (2.10–25.04)**	10.06					
			Zidi S et al (2014)	1.03 (0.64–1.65)	27.67					
			Pandey S et al (2011)	1.00 (0.65–1.54)	29.12					
rs1800629	TNF	Random	Barbisan G et al (2012)	0.60 (0.29–1.24)	11.64	0.05	61.4	0.05	1.22 (0.92–1.63)	0.17
			Ivansson EL et al (2010)	1.14 (0.94–1.39)	36.61					
			Roszak A et al (2015)	1.29 (0.96–1.74)	30.19					
			Sousa H. et al (2014)	**1.87 (1.20–2.91)**	21.56					
rs25487	XRCC1	Random	Wang SS et al (2010)	0.80 (0.61–1.06)	37.82	**0.01**	79.3	0.15	1.09 (0.67–1.79)	0.73
			Roszak A et al (2011)	**1.73 (1.16–2.57)**	33.42					
			Settheetham-Ishida W et al (2011)	0.96 (0.57–1.63)	28.76					

**Table 4:** Meta-analysis for recessive inheritance model of each SNP.

SNP	Gene	Model	Study	OR (95%CI)	Weight (%)	Cochran’s Q	I^2^	Τ^2^	pooled OR (95%CI)	p-value
rs11079454	BRIP1	Fixed	Ma XD et al (2013)	0.79 (0.58–1.08)	52.88	0.99	**0.00**	**0.00**	**0.79 (0.63–0.99)**	**0.04**
			Wang SS et al (2010)	0.81 (0.46–1.41)	15.92					
			Ma XD et al (2013)	0.77 (0.52–1.15)	31.20					
rs2048718	BRIP1	Fixed	Wang SS et al (2010)	0.88 (0.63–1.23)	58.06	0.32	13.1	0.01	1.05 (0.78–1.42)	0.75
			Ma XD et al (2013)	1.35 (0.78–2.34)	26.34					
			Ma XD et al (2013)	1.34 (0.65–2.80)	15.60					
rs6504074	BRIP1	Fixed	Ma XD et al (2013)	0.99 (0.60–1.64)	26.87	0.82	0.00	0.00	1.08 (0.83–1.40)	0.59
			Wang SS et al (2010)	1.15 (0.82–1.63)	58.03					
			Ma XD et al (2013)	0.95 (0.48–1.86)	15.09					
rs231775	CTLA-4	Random	Xioing YH et al (2014)	**0.51 (0.33–0.79)**	21.48	**0.04**	62.8	0.05	0.82 (0.62–1.10)	0.18
			Gokhale P et al (2013)	0.79 (0.45–1.38)	16.18					
			Wang SS et al (2010)	1.10 (0.81–1.50)	28.11					
			Hu L et al (2010)	0.89 (0.72–1.10)	34.23					
rs5742909	CTLA-4	Fixed	Gokhale P et al (2013)	excluded		0.19	41.2	0.54	2.47 (0.55–11.12)	0.24
			Ivansson EL et al (2010)	1.42 (0.44–4.63)	65.49					
			Xioing YH et al (2014)	7.02 (0.84–58.58)	34.51					
rs1800872	IL-10	Random	Wang SS et al (2010)	1.24 (0.79–1.95)	32.77	**<0.01**	83.3	0.24	0.87 (0.47–1.61)	0.66
			Singhal P et al (2014)	**0.46 (0.30–0.72)**	32.98					
			Shekari M et al (2012)	1.15 (0.78–1.72)	34.25					
rs1800896	IL-10	Random	Wang SS et al (2010)	0.80 (0.43–1.48)	25.11	**<0.01**	87.00	0.61	1.51 (0.66–3.45)	0.33
			Wang Q et al (2011)	1.26 (0.68–2.36)	25.05					
			Barbisan G et al (2012)	1.15 (0.50–2.62)	22.62					
			Singhal P et al (2014)	**3.99 (2.65–6.01)**	27.23					
rs16944	IL-1B	Random	Wang SS et al (2010)	1.10 (0.78–1.54)	33.43	0.06	59.9	0.08	1.09 (0.74–1.59)	0.67
			Al-Tahhan MA et al (2011)	**0.34 (0.12–0.98)**	10.14					
			Qian N et al (2010)	1.09 (0.78–1.53)	33.50					
			Zidi S et al (2014)	**1.77 (1.01–3.11)**	22.92					
rs1801133	MTHFR	Fixed	Kohaar I et al (2010)	1.13 (0.30–4.27)	8.93	0.58	0.00	0.00	0.97 (0.65–1.44)	0.87
			Tong SY et al (2011)	1.08 (0.67–1.74)	68.49					
			Mostowska A et al (2011)	0.65 (0.28–1.50)	22.59					
rs11515	p16	Fixed	Thakur N et al (2012)	0.20 (0.01–4.14)	7.43	0.41	0.00	0.00	1.29 (0.56–2.95)	0.55
			Vargas-Torres et al (2014)	1.82 (0.53–6.25)	47.51					
			Wang SS et al (2010)	1.24 (0.37–4.15)	45.06					
rs3088440	p16	Random	Wang SS et al (2010)	0.82 (0.52–1.29)	44.24	**0.04**	68.8	0.47	1.43 (0.56–3.67)	0.45
			Vargas-Torres et al (2014)	1.30 (0.34–4.90)	24.82					
			Thakur N et al (2012)	**3.46 (1.24–9.71)**	30.95					
rs1801270	p21	Fixed	Wang N et al (2013)	**0.62 (0.45–0.87)**	59.37	0.47	**0.00**	**0.00**	**0.69 (0.54–0.89)**	**<0.01**
			Wang SS et al (2010)	0.90 (0.55–1.48)	21.41					
			Jiang P et al (2010)	0.66 (0.37–1.18)	19.21					
rs1042522	p53	Random	Jiang P et al (2010)	**2.01 (1.14–3.57)**	16.45	**<0.01**	91.9	0.74	1.04 (0.50–2.14)	0.92
			Ferreira da Silva I et al (2010)	0.72 (0.42–1.26)	16.56					
			El khair MM et al (2010)	0.92 (0.45–1.87)	15.59					
			Yang SD et al (2014)	**1.62 (1.00–2.61)**	16.99					
			Ye F et al (2010)	**0.30 (0.22–0.43)**	17.63					
			Jiang P et al (2010)	**1.98 (1.18–3.32)**	16.78					
rs1136410	PARP-1	Random	Roszak A et al (2013)	1.54 (0.80–2.97)	28.48	**<0.01**	83.3	0.26	1.61 (0.85–3.04)	0.14
			Ye F et al (2012)	**2.57 (1.85–3.57)**	36.89					
			Wang SS et al (2010)	1.01 (0.66–1.54)	34.63					
rs352140	TLR9	Random	Roszak A et al (2012)	1.19 (0.88–1.62)	38.88	0.06	59.6	0.12	1.39 (0.88–2.19)	0.16
			Lai ZZ et al (2013)	7.07 (0.87–57.54)	4.35					
			Zidi S et al (2014)	**2.02 (1.21–3.40)**	29.45					
			Pandey S et al (2011)	0.88 (0.50–1.56)	27.32					
rs1800629	TNF	Random	Barbisan G et al (2012)	10.34 (0.53–201.98)	7.99	**0.03**	67.5	0.51	1.74 (0.69–4.41)	0.24
			Ivansson EL et al (2010)	0.75 (0.44–1.26)	38.66					
			Roszak A et al (2015)	**2.28 (1.09–4.78)**	34.42					
			Sousa H. et al (2014)	2.81 (0.56–14.06)	18.93					
rs25487	XRCC1	Random	Wang SS et al (2010)	**0.60 (0.37–0.96)**	39.74	**<0.01**	79.2	0.39	0.98 (0.43–2.24)	0.96
			Roszak A et al (2011)	**1.74 (1.07–2.83)**	39.56					
			Settheetham-Ishida W et al (2011)	0.84 (0.22–3.23)	20.70					

Finally, for the allele model only the *rs1801270 p21* gene polymorphism minor allele (*A* allele) had a statistically significant association with CC risk (pooled OR = 0.78; 95%CI: 0.64–0.95; p = 0.01) (Fig 3e), which was in the same direction as the other associations found in the other models. For the BRIP1 polymorphisms minor alleles (*rs2048718* polymorphism *C* allele and *rs11079454* polymorphism *A* allele) we could not detect significant association with CC risk showing a *p* value of 0.12 and 0.19, respectively (Table 5).

**Table 5:** Meta-analysis for minor allele of each SNP.

SNP	Gene	Model	Study	OR (95%CI)	Weight (%)	Cochran’s Q	I^2^	Τ^2^	pooled OR (95%CI)	p-value
rs11079454	BRIP1	Fixed	Ma XD et al (2013)	0.95 (0.79–1.12)	45.29	0.90	0.00	0.00	0.92 (0.82–1.04)	0.19
			Wang SS et al (2010)	0.89 (0.71–1.10)	28.36					
			Ma XD et al (2013)	0.93 (0.74–1.17)	26.34					
rs2048718	BRIP1	Fixed	Wang SS et al (2010)	0.89 (0.74–1.08)	42.98	0.98	0.00	0.00	0.90 (0.80–1.02)	0.12
			Ma XD et al (2013)	0.92 (0.74–1.13)	36.08					
			Ma XD et al (2013)	0.91 (0.69–1.20)	20.93					
rs6504074	BRIP1	Random	Ma XD et al (2013)	0.84 (0.64–1.10)	28.05	0.07	62.2	0.02	0.93 (0.75–1.14)	0.49
			Wang SS et al (2010)	1.12 (0.92–1.36)	36.89					
			Ma XD et al (2013)	0.83 (0.67–1.02)	35.06					
rs231775	CTLA-4	Random	Xioing YH et al (2014)	0.88 (0.71–1.07)	26.30	0.06	59.7	0.02	0.90 (0.76–1.06)	0.20
			Gokhale P et al (2013)	0.69 (0.48–0.97)	15.07					
			Wang SS et al (2010)	1.12 (0.92–1.35)	27.56					
			Hu L et al (2010)	0.86 (0.73–1.01)	31.07					
rs5742909	CTLA-4	Random	Gokhale P et al (2013)	1.01 (0.35–2.94)	9.23	0.06	65.6	0.06	1.28 (0.90–1.82)	0.18
			Ivansson EL et al (2010)	1.07 (0.86–1.33)	47.81					
			Xioing YH et al (2014)	**1.63 (1.24–2.15)**	42.97					
rs1800872	IL-10	Random	Wang SS et al (2010)	1.02 (0.83–1.26)	34.78	**<0.01**	87.8	0.12	0.86 (0.57–1.29)	0.46
			Singhal P et al (2014)	**0.56 (0.44–0.72)**	33.37					
			Shekari M et al (2012)	1.10 (0.82–1.47)	31.85					
rs1800896	IL-10	Random	Wang SS et al (2010)	0.92 (0.73–1.15)	20.52	**<0.01**	93.0	0.25	1.27 (0.80–2.01)	0.30
			Wang Q et al (2011)	1.33 (0.98–1.80)	19.74					
			Barbisan G et al (2012)	1.12 (0.79–1.58)	19.20					
			Singhal P et al (2014)	**2.86 (2.22–3.69)**	20.23					
			Hussain SK et al (2013)	0.85 (0.66–1.09)	20.31					
rs16944	IL-1B	Random	Wang SS et al (2010)	1.05 (0.87–1.27)	30.54	**0.01**	73.2	0.05	1.03 (0.79–1.33)	0.84
			Al-Tahhan MA et al (2011)	**0.50 (0.30–0.84)**	15.14					
			Qian N et al (2010)	**1.22 (1.01–1.48)**	30.37					
			Zidi S et al (2014)	**1.27 (0.93–1.73)**	23.94					
rs1801133	MTHFR	Fixed	Kohaar I et al (2010)	1.04 (0.71–1.52)	23.95	0.66	0.0	0.00	0.97 (0.80–1.16)	0.72
			Tong SY et al (2011)	1.01 (0.77–1.32)	47.74					
			Mostowska A et al (2011)	0.84 (0.59–1.20)	28.31					
rs11515	p16	Random	Thakur N et al (2012)	**0.34 (0.19–0.59)**	29.91	**<0.01**	86.7	0.28	0.78 (0.41–1.48)	0.45
			Vargas-Torres et al (2014)	1.06 (0.73–1.52)	34.66					
			Wang SS et al (2010)	1.18 (0.86–1.64)	35.44					
rs3088440	p16	Fixed	Wang SS et al (2010)	**0.99 (0.80–1.22)**	49.80	0.25	27.1	0.01	1.09 (0.90–1.32)	0.39
			Vargas-Torres et al (2014)	1.02 (0.71–1.47)	22.50					
			Thakur N et al (2012)	1.36 (0.99–1.88)	27.70					
rs1801270	p21	Random	Wang N et al (2013)	**0.70 (0.58–0.86)**	40.24	0.13	51.7	0.02	**0.78 (0.64–0.95)**	**0.01**
			Wang SS et al (2010)	0.93 (0.75–1.14)	37.96					
			Jiang P et al (2010)	**0.69 (0.48–0.98)**	21.79					
rs1042522	p53	Random	Jiang P et al (2010)	**1.50 (1.06–2.13)**	13.99	**<0.01**	78.3	0.09	1.16 (0.90–1.49)	0.26
			Ferreira da Silva I et al (2010)	1.05 (0.76–1.45)	14.60					
			El khair MM et al (2010)	0.82 (0.56–1.20)	13.31					
			Yang SD et al (2014)	**1.56 (1.12–2.18)**	14.39					
			Ye F et al (2010)	**0.79 (0.67–0.92)**	17.73					
			Djansugurova LB et al (2013)	1.34 (0.88–2.04)	12.57					
			Jiang P et al (2010)	**1.46 (1.00–2.14)**	13.42					
rs1136410	PARP-1	Random	Roszak A et al (2013)	**1.38 (1.08–1.76)**	30.94	**<0.01**	83.2	0.05	1.18 (0.90–1.57)	0.23
			Ye F et al (2012)	**1.35 (1.16–1.58)**	35.78					
			Wang SS et al (2010)	0.89 (0.73–1.09)	33.28					
rs352140	TLR9	Fixed	Roszak A et al (2012)	**1.33 (1.10–1.61)**	23.27	0.11	46.9	0.01	1.09 (0.96–1.25)	1.18
			Wang SS et al (2010)	0.91 (0.75–1.10)	23.20					
			Chen X et al (2012)	1.10 (0.95–1.29)	28.50					
			Bodelon C et al (2010)	1.07 (0.90–1.28)	24.51					
			Lai ZZ et al (2013)	1.25 (0.21–7.58)	0.52					
rs352140	TLR9	Random	Roszak A et al (2012)	**1.20 (1.00–1.45)**	25.65	<0.01	78.6	0.06	1.19 (0.91–1.56)	0.20
			Lai ZZ et al (2013)	**7.00 (2.42–20.23)**	5.28					
			Zidi S et al (2014)	1.31 (0.96–1.80)	20.94					
			Bodelon C et al (2010)	0.91 (0.77–1.09)	25.93					
			Pandey S et al (2011)	0.97 (0.73–1.28)	22.20					
rs1800629	TNF	Random	Barbisan G et al (2012)	0.79 (0.41–1.52)	10.87	0.06	60.00	0.04	1.23 (0.96–1.58)	0.10
			Roszak A et al. (2015)	1.33 (1.04–1.70)	31.16					
			Ivansson EL et al (2010)	1.08 (0.90–1.28)	37.23					
			Sousa H. et al (2014)	1.78 (1.19–2.68)	20.74					
rs25487	XRCC1	Random	Wang SS et al (2010)	0.78 (0.64–0.98)	29.27	<0.01	79.7	0.10	1.09 (0.77–1.55)	0.61
			Djansugurova LB et al (2013)	1.30 (0.84–2.00)	21.61					
			Roszak A et al (2011)	**1.49 (1.15–1.93)**	27.63					
			Settheetham-Ishida W et al (2011)	0.96 (0.62–1.48)	21.48					
rs1800871	IL-10	Fixed	Singhal P et al (201	**1.24 (0.97–1.58)**	31.80	0.43	0.00	0.00	1.08 (0.94–1.24)	0.27
			Wang SS et al (2010)	1.02 (0.83–1.26)	45.66					
			Hussain SK et al (2013)	**1.00 (0.75–1.34)**	22.53					
rs861539	XRCC3	Random	Wang SS et al (2010)	0.96 (0.75–1.22)	36.25	0.04	63.1	0.07	1.04 (0.73–1.48)	0.82
			Settheetham-Ishida W. et al (2011)	0.88 (0.37–2.08)	12.12					
			Djansugurova LB et al (2013)	**2.04 (1.16–3.60)**	20.49					
			Pérez LO et al (2013)	0.79 (0.56–1.10)	31.13					
rs9344	CCND1	Random	Djansugurova LB et al (2013)	0.97 (0.65–1.46)	29.42	**<0.01**	84.2	0.12	1.02 (0.67–1.56)	0.92
			Warchol T et al (2011)	**1.46 (1.09–1.97)**	33.73					
			Wang N et al (2012)	**0.76 (0.62–0.94)**	36.85					

**Fig 4:**
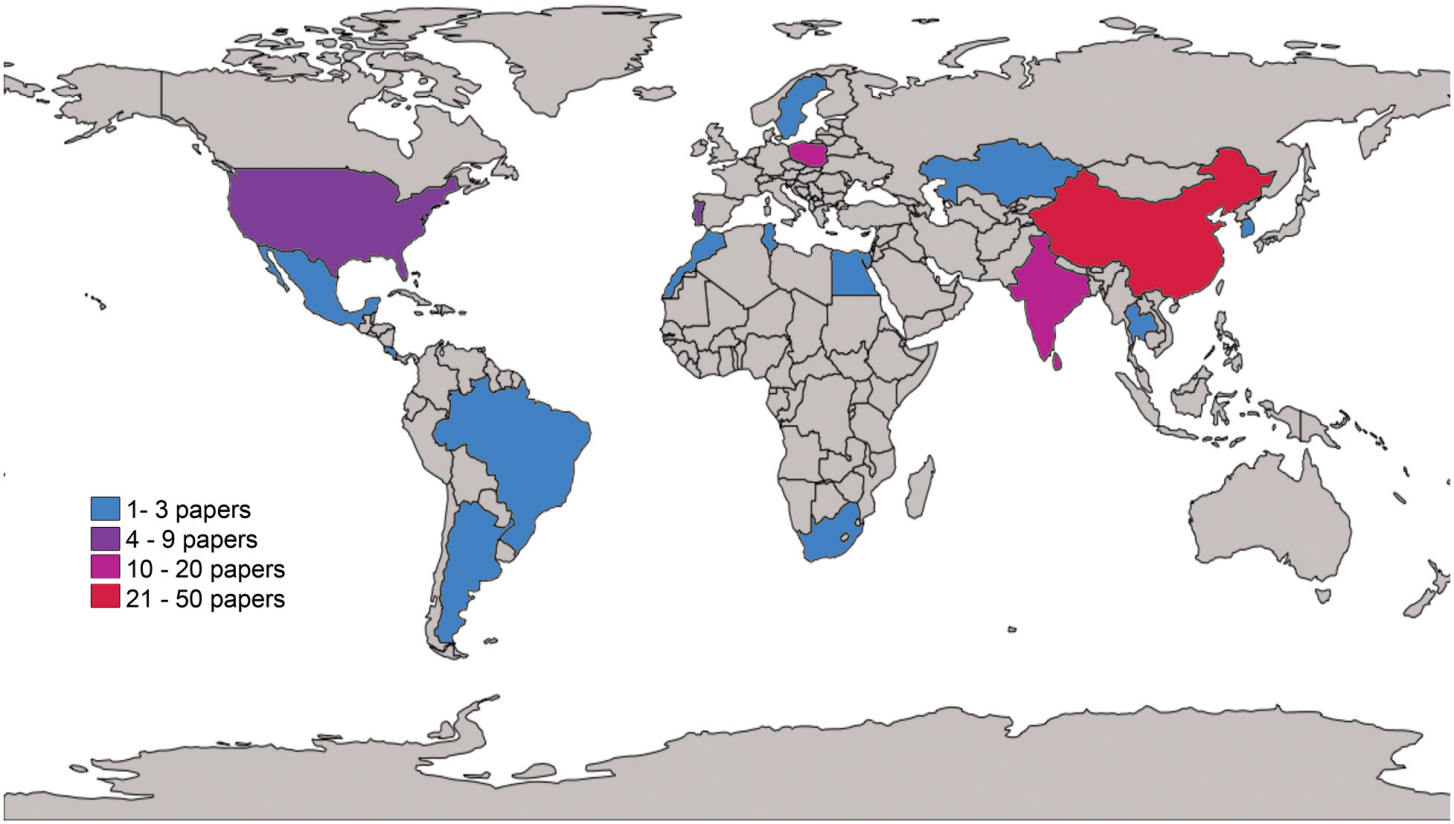
Number of studies reviewed by country.

Given that the number of studies per polymorphism was so limited, we could not perform meta-analyses stratifying by ethnicity. More than half (67%) of the studies included in the review were from an Asiatic population, 17% from a Caucasian population and only 4% from an African population. In fact, the median of the number of studies included in the review was of two studies per country, with an interquartile range of three studies. The country with most studies included was China with 44 studies, followed by India with 11 studies and Poland with 10 studies (Table 6 and Fig 4).

**Table 6:** Number of studies reviewed by country.

Country	Genotypic models		Allelic model	
	N	%	N	%
Argentina	2	2.11	2	2.00
Brazil	2	2.11	2	2.00
China	44	46.32	46	46.00
Costa Rica	2	2.11	2	2.00
Egypt	1	1.05	1	1.00
India	11	11.58	11	11.00
Kazakhstan	--	--	1	1.00
Korea	3	3.16	3	3.00
Mexico	2	2.11	2	2.00
Morocco	1	1.05	1	1.00
Poland	10	10.53	10	10.00
Portugal	4	4.21	4	4.00
South Africa	1	1.05	1	1.00
Sweden	3	3.16	3	3.00
Taiwan	4	4.21	4	4.00
Thailand	2	2.11	2	2.00
Tunisia	1	1.05	1	1.00
United States	2	2.11	4	4.00
TOTAL	95	100	100	100

**Fig 5:**
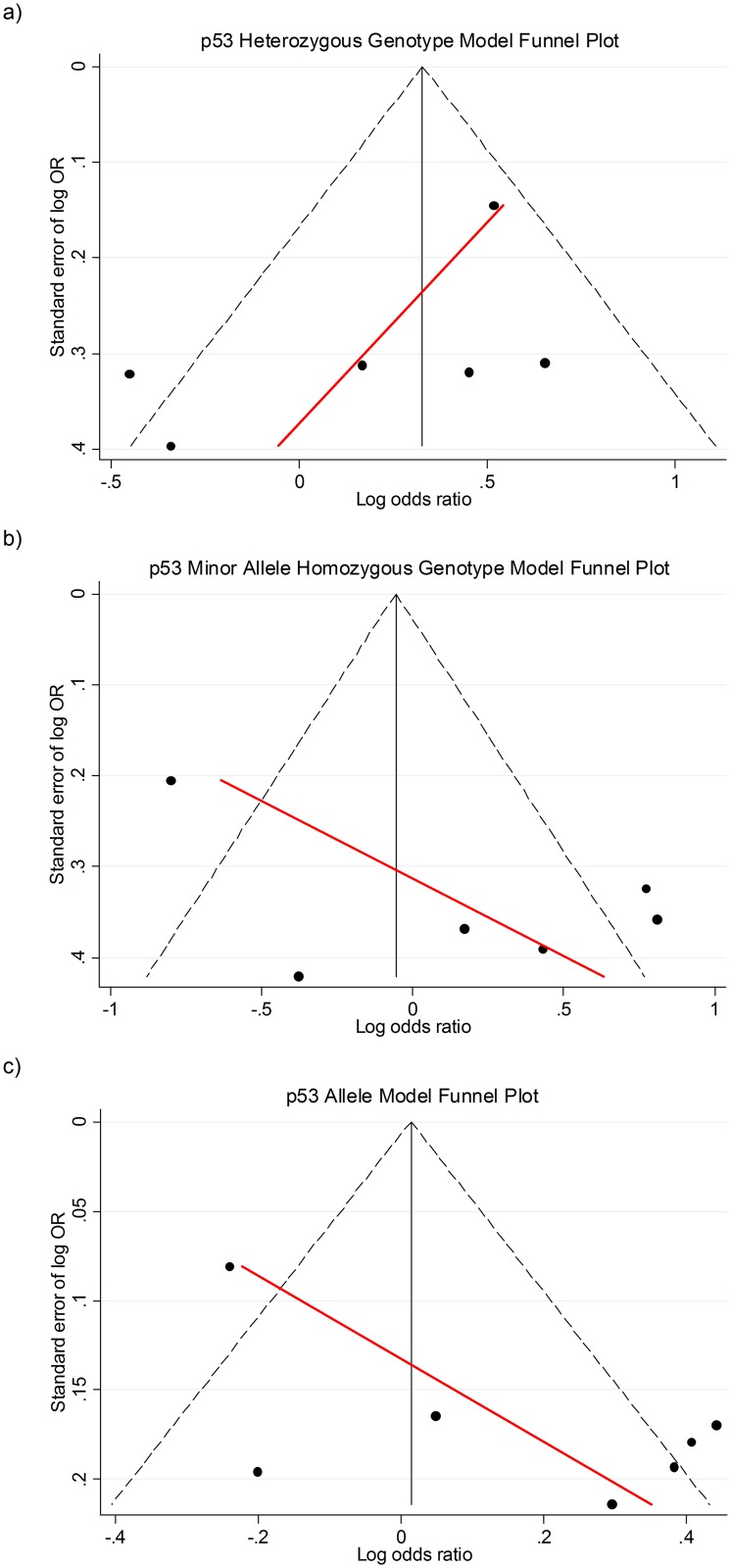
Egger test for p53 rs1042522 polymorphism a) heterozygous, b) minor allele homozygous and c) allele models. The red solid line in each funnel-plot represents the 95% confidence limits around the summary polymorphism effect.

Only the *p53 rs1042522* polymorphism had the minimum five studies required to assess the possibility of publication bias. The Egger test for the heterozygous, minor allele homozygous and allele models are presented as funnel-plots in Fig 5.

## Discussion

The main findings of our study were a significant negative association between the *p21* gene polymorphism (*rs1801270*), two *BRIP1* gene polymorphisms (*rs2048718* and *rs11079454*) and CC. Carriers of one or two copies of the minor allele (dominant inheritance model, *C/A + A/A*) of the *p21* gene polymorphism (*rs1801270*) were 32% less likely to develop CC than carriers of the homozygous genotype for the ancestral allele (*C/C*). Likewise, carriers of at least one copy of the *rs2048718 BRIP1* gene polymorphism minor allele (dominant inheritance model, *T/C + C/C*) were 20% less likely to develop CC than carriers of the homozygous genotype for the ancestral allele (T/T). We only found association with the recessive inheritance model for the *rs11079454* polymorphism of this gene, in which the carriers of two copies of the minor allele (*A/A*) were 27% less likely to develop CC than carriers of one or two ancestral alleles (*T/A + T/T*).

To our knowledge, to date only two previous studies of extensive meta-analysis of genetic polymorphisms and CC have been made [13,21]. Unlike these studies, our study was limited to studies of associations of gene polymorphisms and CC risk published in English, Spanish or Portuguese in the last five years, in order to seek more uniformity in polymorphism genotyping technologies used in the included studies. Additionally, we concentrated our analysis on case-control studies and did not include studies with other designs, such as cross-sectional and cohort studies as the one carried out by Zhang *et al*, 2014 [13]. Furthermore, given the high reproducibility of HLA genetic markers association with CC, our analysis did not focus on HLA antigen genetic markers as the study of Wang *et al*, 2015 [21].

Since there was a limited number of studies by polymorphism, it was not possible to analyze ethnic subgroups, as performed by Wang *et al*, 2015 [21]. In contrast to our study, the divergence in the SNP reported as significantly associated with risk of developing CC in the previous two studies may be due to the inclusion criteria defined. On the contrary, Zhang *et al*, 2014 [13] and Wang *et al*, 2015[21], did not find significant negative associations between any SNP and CC. Paradoxically, the *p21* gene polymorphism (*rs1801270*) which we consistently reported as a protective factor for CC in our study was not found to be significantly associated with CC by any of these authors.

The *p21* gene (also known as *CDKN1A*) encodes a 21-kda protein and was first described as a potent inhibitor of cell proliferation and DNA replication, both in physiological conditions and after DNA damage [22]. As the main downstream regulator of tumor suppressor *p53*, *p21* functions as a unique link between p53, cell-cycle arrest and DNA repair, exhibiting anti-oncogenic properties [23,24].

On the other hand, studies focused on the role of p21 in ATM-mediated signal pathways have shown that p21 is a downstream effector of ATM-mediated growth control, and have suggested that loss pf p21 in *Atm*-deficient mice results in a delay in thymic lymphomagenesis and in an apparent increase in spontaneous apoptosis in tumor cells [25,26]. This indicates that the loss of p21 function might have tumor suppression consequences. Additionally, the evidence generated from p21 knockout models and expression patterns in human cancer samples shows the duality of its role in carcinogenesis, playing a role as both tumor suppressor and oncogene [27].

Several studies have suggested that *p21* polymorphisms affect protein expression and activity, and hence might play a role in susceptibility to cancer [27,28]. In that sense, a relatively large number of studies have evaluated the association between *p21* rs1801270 (*Ser*31*Arg*) polymorphism and the risk of several types of cancers (lung cancer, breast cancer, CC, gastric cancer, etc.), though the results remain inconclusive [23,29–31] due to the complexity of p21 function.

*p21* rs1801270 polymorphism produces a *C* to *A* transversion and causes a substitution from serine (*Ser*) to arginine (*Arg*), affecting the DNA binding zinc finger domain of the protein [32]. Interestingly, transfection studies have demonstrated that the expression of the *A* allele of this polymorphism does not affect the previously reported tumor suppressor activity of p21 [33]. However, the molecular mechanism underlying the protective effect of the rs1801270 *A* allele (allele codifying an arginine) in CC is still uncertain.

A previous meta-analysis focused only on the *p21 rs1801270* polymorphism and gastrointestinal tract tumor risk reported that the ancestral allele (*C* allele) might be a risk factor for this type of cancer among Asiatic women [23]. Furthermore, this finding was also reported in a meta-analysis focused on breast cancer risk among Caucasians [34]. In that sense, our study is consistent with the reported by Dong Y *et al*. [23] and Qiu L *et al*. [34], since we detected that the ancestral allele (*C* allele) might be a risk for CC. Although in previous meta-analyses centered on CC risk this polymorphism was not reported as significantly associated, we believe that the environmental and gene interactions have an important role over the effect of this SNP over CC development, given the complexity and duality of its role over oncogenesis.

The *BRIP1* gene codifies a DNA-dependent ATPase and a DNA helicase denominated BRCA1-interacting protein 1, which belongs to the RecQ DEAH helicase family [35]. Since it is critical for the BRCA-associated DNA damage repair process the *BRIP1* gene has been associated with other types of cancer, especially breast cancer [35–38].

Particularly for CC, *BRIP1* was found to be overexpressed in advanced squamous cervical cancer biopsies of non-responsive to base-line therapy patients, as compared to biopsies of responsive patients[39]. However, there are few reports that assess the potential role of genetic markers in different regions of the *BRIP1* gene in the risk of CC [40,41].

Both of the *BRIP1* polymorphisms we found to be associated with CC risk fall in the untranslated region (UTR) of the gene. The *rs2048718 BRIP1* polymorphism is located in the 5´-UTR, whereas the *rs11079454* polymorphism is in the 3´-UTR.

Assessing the functionality of polymorphisms located in the UTR is quite a challenge and, even though there are no reports of the direct effect *rs2048718* or *rs11079454 BRIP1* polymorphisms have in CC development, there is evidence suggesting that at least *rs11079454* might be affecting the expression of the *BRIP1* gene [37,41]. The *rs11079454* is in LD (linkage disequilibrium) with other three *BRIP1* SNP (*rs7213430*, *rs4986763*, *rs11871785*), and the haplotype of this LD block including the ancestral allele (*T* allele) of *rs11079454* polymorphism was associated with CC risk among Chinese women, which is consistent with the association observed in this meta-analysis [40].

The meta-analysis at hand has limitations, and the interpretation of the results presented here should be made in the context of these. In the first instance, to avoid the potential bias of genotyping inaccuracy given by the genotyping method used in the studies, we included only recent studies which limited the number of studies per polymorphism. The reduced number of reports per SNP included affected the statistical power of the study to detect small effects. Additionally, this affected the assessment of potential publication bias, as it was only possible to evaluate it in the *p53 rs1042522* polymorphism. The shortage of studies per polymorphism could be explained by the fact that GWAS (Genome-wide association studies) increasingly reveal new variants associated with CC, but replication studies in different populations for these variants are scarce. It is, therefore, difficult to conclude the true role of such variants in CC susceptibility. There is a clear need for replication studies that can validate the association of the GWAS discovered genetic variants and reveal their role in genetic susceptibility to CC in each population. Similarly, studies from several candidate genes on their own do not provide conclusive and reproducible data for other populations, despite reporting statistically significant associations. Finally, we cannot guarantee that population stratification did not affect the results of the constituent studies in the meta-analyses, since we could not perform an analysis stratified by ethnicity.

From a wider perspective, lack of reproducibility of the candidate SNP studies is unfortunately a general trend beyond CC. The boom of GWAS generated a global effort to study a large number of SNP without a clear path nor a previous biological hypothesis, leading to a picking and fishing approach. This practice was acceptable in the beginning of the genomic age but nowadays a systematic effort that guarantees reproducibility is highly required [42].

## Conclusions

Notwithstanding the presence of heterogeneity due to factors not addressed in this study, our results provide evidence of the negative association between *p21* rs1801270 polymorphism, as well as BRIP1 rs2048718 and rs11079454 polymorphisms, and CC risk. Additionally, this study highlights the urgent need for more replication studies focused on GWAS identified CC susceptibility variants.

## Supporting Information

S1 File PRISMA guidelines checklist. (PDF)

S2 File PLOS ONE Meta-analysis on genetic association studies checklist. (DOCX)

S1 Table Data from the case-control articles included in the meta-analysis. (XLS)

S2 Table Data from the articles excluded in the meta-analysis. (XLSX)

S3 Table Median and interquartile range of the number of reports per SNP reviewed. (XLSX)

## References

[1] Ferlay J, Shin H-R, Bray F, Forman D, Mathers C, Parkin DM. Estimates of worldwide burden of cancer in 2008: GLOBOCAN 2008. Int J Cancer. 2010;127: 2893–917. 10.1002/ijc.25516 21351269

[2] Arbyn M, Castellsagué X, de Sanjosé S, Bruni L, Saraiya M, Bray F, et al Worldwide burden of cervical cancer in 2008. Ann Oncol. 2011;22: 2675–86. 10.1093/annonc/mdr015 21471563

[3] Dirección General de Epidemiología, Secretaría de Salud de México. Anuario de Morbilidad 1984–2014. [Internet]. [cited 13 May 2016]. Available: http://www.epidemiologia.salud.gob.mx/anuario/20140/morbilidad/enfermedad/distribucion_casos_nuevos_enfermedad_grupo_edad.pdf

[4] IARC/WHO. GLOBOCAN 2012: Estimated cancer incidence, mortality and prevalence Worldwide in 2012 [Internet]. [cited 12 Oct 2015]. Available: http://globocan.iarc.fr/Pages/fact_sheets_population.aspx

[5] Bosch FX. Human papillomavirus: science and technologies for the elimination of cervical cancer. Expert Opin Pharmacother. 2011;12: 2189–204. 10.1517/14656566.2011.596527 21756205

[6] Secretaria de Salud de México. Modificación a la Norma Oficial Mexicana NOM-014-SSA2-1994, Para la prevención, detección, diagnóstico, tratamiento, control y vigilancia epidemiológica del cáncer cérvico uterino. [Internet]. [cited 13 May 2016]. Available: http://www.salud.gob.mx/unidades/cdi/nom/m014ssa294.pdf

[7] Torres-Poveda K, Cruz-Valdez A, Madrid-Marina V. Epidemiología del Cáncer cervicouterino. Gac Mex Oncol. 2014;13: 4–17.

[8] Torres-Poveda K, Burguete-García AI, Bahena-Román M, Méndez-Martínez R, Zurita A, López-Estrada G, et al Risk allelic load in Th2 and Th3 cytokines genes as biomarker of susceptibility to HPV-16 positive cervical cancer: a case control study. BMC Cancer. 2016;16(1):330 10.1186/s12885-016-2364-4 27220278 PMC4879749

[9] Audirac-Chalifour A, Torres-Poveda K, Bahena-Román M, Téllez-Sosa J, Martínez-Barnetche J, Cortina-Ceballos B, et al Cervical Microbiome and Cytokine Profile at Various Stages of Cervical Cancer: A Pilot Study. PLoS One. 2016;11: e0153274 10.1371/journal.pone.0153274 27115350 PMC4846060

[10] Stanley MA, Sterling JC. Host responses to infection with human papillomavirus. Curr Probl Dermatol. 2014;45: 58–74. 10.1159/000355964 24643178

[11] Song D, Li H, Li H, Dai J. Effect of human papillomavirus infection on the immune system and its role in the course of cervical cancer. Oncol Lett. 2015;10: 600–606. 10.3892/ol.2015.3295 26622540 PMC4509451

[12] Torres-Poveda K, Bahena-Román M, Madrid-González C, Burguete-García AI, Bermúdez-Morales VH, Peralta-Zaragoza O, et al Role of IL-10 and TGF-β1 in local immunosuppression in HPV-associated cervical neoplasia. World J Clin Oncol. 2014;5: 753–63. 10.5306/wjco.v5.i4.753 25302175 PMC4129538

[13] Zhang X, Zhang L, Tian C, Yang L, Wang Z. Genetic variants and risk of cervical cancer: epidemiological evidence, meta-analysis and research review. BJOG. 2014;121: 664–74. 10.1111/1471-0528.12638 24548744

[14] Moher D, Liberati A, Tetzlaff J, Altman DG. Preferred reporting items for systematic reviews and meta-analyses: the PRISMA statement. PLoS Med. Public Library of Science; 2009;6: e1000097 10.1371/journal.pmed.1000097 PMC2707599 19621072

[15] Huedo-Medina TB, Sánchez-Meca J, Marín-Martínez F, Botella J. Assessing heterogeneity in meta-analysis: Q statistic or I^2^ index? Psychol Methods. 2006;11: 193–206. 16784338 10.1037/1082-989X.11.2.193

[16] Bedi U, Singh M, Singh P, Molnar J, Khosla S, Arora R. Effects of statins on progression of coronary artery disease as measured by intravascular ultrasound. J Clin Hypertens (Greenwich). 2011;13: 492–6. 10.1111/j.1751-7176.2011.00428.x 21762362 PMC8108916

[17] Egger M, Davey Smith G, Schneider M, Minder C. Bias in meta-analysis detected by a simple, graphical test. BMJ. 1997;315: 629–34. 9310563 10.1136/bmj.315.7109.629 PMC2127453

[18] Sterne JAC, Becker B, Egger M. The funnel plot In: Rothstein HR, Sutton AJ, Borenstein M, editors. Publication Bias in Meta-Analysis Prevention, Assessment and Adjustments. first. Manchester: John Wiley & Sons Ltd, The Atrium, Southern Gate, Chichester; 2005 pp. 75–98.

[19] Hedges L V, Pigott TD. The power of statistical tests in meta-analysis. Psychol Methods. 2001;6: 203–217. 10.1037/1082-989X.6.3.203 11570228

[20] Gelman A. Commentary: P Values and Statistical Practice. Epidemiology. 2013;24: 69–72. 23232612 10.1097/EDE.0b013e31827886f7

[21] Wang S, Sun H, Jia Y, Tang F, Zhou H, Li X, et al Association of 42 SNPs with genetic risk for cervical cancer: an extensive meta-analysis. BMC Med Genet. 2015;16: 1–10. 25928231 10.1186/s12881-015-0168-z PMC4436168

[22] Xiong Y, Hannon GJ, Zhang H, Casso D, Kobayashi R, Beach D. p21 is a universal inhibitor of cyclin kinases. Nature. 1993;366: 701–4. 10.1038/366701a0 8259214

[23] Dong Y, Wang X, Ye X, Wang G, Li Y, Wang N, et al Association Between p21 Ser31Arg Polymorphism and Gastrointestinal Tract Tumor Risk: A Meta-analysis. Technol Cancer Res Treat. 2015;14: 627–33. 10.7785/tcrtexpress.2013.500422 24645745 PMC4639905

[24] Fotedar R, Bendjennat M, Fotedar A. Role of p21WAF1 in the cellular response to UV. Cell Cycle. 2004;3: 134–7. 14712074

[25] Wang YA, Elson A, Leder P. Loss of p21 increases sensitivity to ionizing radiation and delays the onset of lymphoma in atm-deficient mice. Proc Natl Acad Sci U S A. 1997;94: 14590–5. 9405657 10.1073/pnas.94.26.14590 PMC25064

[26] Shao C, Liang L, Zhao X, Chen Y, Zheng B, Chen J, et al Mutagenesis in vivo in T cells of p21-deficient mice. Mutat Res. 2009;670: 103–6. 10.1016/j.mrfmmm.2009.09.001 19744501 PMC2767417

[27] Roninson IB. Oncogenic functions of tumour suppressor p21(Waf1/Cip1/Sdi1): association with cell senescence and tumour-promoting activities of stromal fibroblasts. Cancer Lett. 2002;179: 1–14. 11880176 10.1016/s0304-3835(01)00847-3

[28] Li G, Liu Z, Sturgis EM, Shi Q, Chamberlain RM, Spitz MR, et al Genetic polymorphisms of p21 are associated with risk of squamous cell carcinoma of the head and neck. Carcinogenesis. 2005;26: 1596–602. 10.1093/carcin/bgi105 15878916

[29] Li Y, Liu F, Tan S, Li S. P21 Ser31Arg polymorphism and cervical cancer risk: a meta-analysis. Int J Gynecol Cancer. 2011;21: 445–51. 10.1097/IGC.0b013e31820da58b 21430453

[30] Ma Y, Zhang Y, Lin L, Guo X, Wu Y, Wen W, et al Quantitative assessment of the relationship between p21 Ser31Arg polymorphism and cervical cancer. Tumour Biol. 2013;34: 3887–92. 10.1007/s13277-013-0976-8 23832542

[31] Wang N, Wang S, Zhang Q, Lu Y, Wei H, Li W, et al Association of p21 SNPs and risk of cervical cancer among Chinese women. BMC Cancer. 2012;12: 589 10.1186/1471-2407-12-589 23231583 PMC3527144

[32] Sun Y, Hildesheim A, Li H, Li Y, Chen JY, Cheng YJ, et al No point mutation but a codon 31ser—>arg polymorphism of the WAF-1/CIP-1/p21 tumor suppressor gene in nasopharyngeal carcinoma (NPC): the polymorphism distinguishes Caucasians from Chinese. Cancer Epidemiol Biomarkers Prev. 4: 261–7. 7606201

[33] Chedid M, Michieli P, Lengel C, Huppi K, Givol D. A single nucleotide substitution at codon 31 (Ser/Arg) defines a polymorphism in a highly conserved region of the p53-inducible gene WAF1/CIP1. Oncogene. 1994;9: 3021–4. 8084608

[34] Qiu L-X, Zhang J, Zhu X-D, Zheng C-L, Sun S, Wang Z-H, et al The p21 Ser31Arg polymorphism and breast cancer risk: a meta-analysis involving 51,236 subjects. Breast Cancer Res Treat. 2010;124: 475–9. 10.1007/s10549-010-0858-3 20349127

[35] Ouhtit A, Gupta I, Shaikh Z. BRIP1, a potential candidate gene in development of non-BRCA1/2 breast cancer. Front Biosci (Elite Ed). 2016;8: 289–98. 26709662 10.2741/E767

[36] Norquist BM, Harrell MI, Brady MF, Walsh T, Lee MK, Gulsuner S, et al Inherited Mutations in Women With Ovarian Carcinoma. JAMA Oncol. 2015; 1–9. 10.1001/jamaoncol.2015.5495 PMC4845939 26720728

[37] Liu H, Gao F, Dahlstrom KR, Li G, Sturgis EM, Zevallos JP, et al A variant at a potentially functional microRNA-binding site in BRIP1 was associated with risk of squamous cell carcinoma of the head and neck. Tumour Biol. 2015; 10.1007/s13277-015-4682-6 PMC4958391 26711789

[38] Pabalan N, Jarjanazi H, Ozcelik H. Association between BRIP1 (BACH1) polymorphisms and breast cancer risk: a meta-analysis. Breast Cancer Res Treat. 2013;137: 553–8. 10.1007/s10549-012-2364-2 23225146

[39] Balacescu O, Balacescu L, Tudoran O, Todor N, Rus M, Buiga R, et al Gene expression profiling reveals activation of the FA/BRCA pathway in advanced squamous cervical cancer with intrinsic resistance and therapy failure. BMC Cancer. 2014;14: 246 10.1186/1471-2407-14-246 24708616 PMC4021393

[40] Ma XD, Cai GQ, Zou W, Huang YH, Zhang JR, Wang DT, et al First evidence for the contribution of the genetic variations of BRCA1-interacting protein 1 (BRIP1) to the genetic susceptibility of cervical cancer. Gene. 2013;524: 208–13. 10.1016/j.gene.2013.04.025 23644138

[41] Ma XD, Cai GQ, Zou W, Huang YH, Zhang JR, Wang DT, et al BRIP1 variations analysis reveals their relative importance as genetic susceptibility factor for cervical cancer. Biochem Biophys Res Commun. 2013;433: 232–6. 10.1016/j.bbrc.2013.02.089 23473757

[42] Ward LD, Kellis M. Interpreting noncoding genetic variation in complex traits and human disease. Nat Biotechnol. 2012;30: 1095–106. 10.1038/nbt.2422 23138309 PMC3703467

